# Amyloid imaging for differential diagnosis of dementia: incremental value compared to clinical diagnosis and [^18^F]FDG PET

**DOI:** 10.1007/s00259-018-4111-3

**Published:** 2018-08-10

**Authors:** Sabine Hellwig, Lars Frings, Tobias Bormann, Werner Vach, Ralph Buchert, Philipp T. Meyer

**Affiliations:** 1 Department of Psychiatry and Psychotherapy, Medical Center – University of Freiburg, Faculty of Medicine, University of Freiburg, Hauptstr. 5, 79104 Freiburg, Germany; 2 Department of Nuclear Medicine, Medical Center – University of Freiburg, Faculty of Medicine, University of Freiburg, Freiburg, Germany; 3 Center for Geriatrics and Gerontology Freiburg, Medical Center – University of Freiburg, Faculty of Medicine, University of Freiburg, Freiburg, Germany; 4 Department of Neurology, Medical Center – University of Freiburg, Faculty of Medicine, University of Freiburg, Freiburg, Germany; 5 Clinical Epidemiology, Institute of Medical Biometry and Medical Informatics, University of Freiburg, Freiburg, Germany; 6 Department of Orthopaedics and Traumatology, University Hospital Basel, Basel, Switzerland; 7 Department of Diagnostic and Interventional Radiology and Nuclear Medicine, University Hospital Hamburg Eppendorf, Hamburg, Germany

**Keywords:** Positron emission tomography, [^11^C]PIB, [^18^F]FDG, Amyloid imaging, Dementia

## Abstract

**Purpose:**

Cerebral beta-amyloid and regional glucose metabolism assessed by positron emission tomography (PET) are used as diagnostic biomarkers for Alzheimer’s disease (AD). The present study validates the incremental diagnostic value of amyloid PET in addition to clinical diagnosis and [^18^F]FDG PET in a real-life memory clinic population.

**Methods:**

Of 138 consecutive patients with cognitive impairment who received combined [^18^F]FDG and [^11^C]PIB PET, 84 were diagnosed with major neurocognitive disorder (DSM-5) and included. Baseline clinical and [^18^F]FDG PET diagnoses were independently established with and without access to amyloid PET results and were dichotomized into AD or non-AD disorders. The incremental value of amyloid PET was evaluated in terms of: (1) the change in clinical and [^18^F]FDG PET diagnoses, (2) the change in agreement between clinical and [^18^F]FDG PET diagnoses, and (3) diagnostic accuracy using an interdisciplinary consensus diagnosis after an extended follow-up (2.4 ± 1.3 years after PET) as the reference.

**Results:**

After disclosure of the amyloid PET results, clinical and [^18^F]FDG PET diagnoses changed in 23% and 18% of patients, respectively, and agreement between both ratings increased from 62% to 86% (*p* < 0.001). The accuracy of clinical and [^18^F]FDG PET diagnoses improved from 71% to 89% (*p* < 0.01) and from 76% to 94% (*p* < 0.001), respectively. The additional value of amyloid PET was rather uniform in relation to age at onset and consistency with appropriate use criteria.

**Conclusion:**

Amyloid PET provides significant incremental diagnostic value beyond clinical and [^18^F]FDG PET diagnoses of AD. Given the high diagnostic accuracy of combined clinical and amyloid PET assessment, further studies are needed to clarify the role of an additional [^18^F]FDG PET scan in these patients.

## Introduction

Early and correct differential diagnosis of dementia is of therapeutic and prognostic importance. However, the clinical diagnosis is of limited accuracy. A multicentre post-mortem study [1] yielded a sensitivity and specificity of only 71% each for a clinical diagnosis of ‘probable Alzheimer’s disease (AD)’ and of 83% and 55% for ‘possible AD’, respectively, when the clinical diagnosis was based on the 1984 criteria of McKhann et al. [2]. According to the revised criteria [3], imaging of regional cerebral glucose metabolism (with [^18^F]FDG) and of cerebral beta-amyloid deposits with positron emission tomography (PET) may be employed to enhance diagnostic certainty. In the differential diagnostics of AD, the two approaches are complementary: [^18^F]FDG PET detects neurodegeneration and amyloid imaging detects underlying AD pathology. Thus, a combined assessment of neurodegeneration and amyloid pathology is proposed in the guidelines of McKhann et al. [3] and is often performed in clinical routine to achieve optimal diagnostic confidence. On the other hand, effective PET imaging is desirable to minimize patient discomfort, costs, delay in diagnosis and radiation burden.

In 2012, the US Food and Drug Administration and the European Medicines Agency approved amyloid imaging probes (^18^F-labelled tracers), which have become commercially available. Several recent studies have examined the clinical impact of amyloid PET in terms of changes in diagnosis, diagnostic confidence and treatment. Focusing on larger (more than 50 patients) or prospective investigations, these studies yielded fairly consistent results: At variance with the baseline diagnosis (i.e. without knowledge of the amyloid PET findings), 16–39% of subjects with suspected AD were found be amyloid-negative. In turn, a slightly larger fraction of patients (29% to 57%) with a baseline diagnosis of cognitive impairment not due to AD (non-AD) were amyloid-positive [4–10]. This translates into a change in diagnoses in about 20–30% (range 9–67%) of all patients [4–16], in which the lower and upper ranges of observed frequencies of change were found in patients with early onset or from research populations (few uncertain diagnoses) [4, 16] and in patients with late onset or a particularly challenging diagnosis [4, 14–16], respectively.

Given that non-AD cognitive impairment may be associated with an age-dependent increase of concomitant amyloid pathology, whereas an amyloid-negative scan strongly argues against AD as the cause of cognitive impairment (e.g. [17]), it is comprehensible that a negative amyloid scan was more often associated with a change in diagnosis than a positive amyloid scan in some but not all studies [5, 12–14]. As would be expected, changes in diagnosis were accompanied by significant increases in diagnostic confidence [5–11, 14] and changes in patient management in most studies, albeit to a variable extent (about 40–90%) depending on the items under consideration (e.g. medication, diagnostic tests, referrals, counselling) [5, 7, 10, 11, 14]. In this regard, a particularly interesting study is the recent multicentre, randomized controlled study by Pontecorvo et al. [9]. They found significantly higher proportions of patients with a change in diagnosis (33% vs. 6%) and treatment (68% vs. 56%) in the intervention arm compared with the control arm (618 subjects 1:1 randomized to immediate or delayed disclosure of amyloid PET results).

The majority of the studies discussed above predominantly or exclusively included patients from a specialized memory clinic setting with relevant uncertainty regarding the cause of cognitive impairment, early-onset AD (≤65 years) or atypical AD features. Thus, the majority of patients implicitly or sometimes explicitly fulfilled the Appropriate Use Criteria (AUC) of the Amyloid Imaging Task Force [18]. However, to the best of our knowledge, only two studies have directly addressed the impact of consistency with the AUC. In a limited sample of 28 patients with late-onset cognitive impairment, Apostolova et al. [4] observed more frequent diagnostic changes in the AUC-consistent group (53%) than the AUC-inconsistent group (23%), but the difference did not reach the level of significance. In contrast, Grundman et al. [15] found significantly higher proportion of patients with a change in diagnosis in the AUC-consistent group (62%; *n* = 124) than in the AUC-inconsistent group (45%; *n* = 104).

[^18^F]FDG PET was used for diagnostic work-up to a variable extent in the studies discussed above. In four studies, most of the patients also underwent [^18^F]FDG PET. Two studies showed a diagnostic impact of amyloid PET in patients with an uncertain diagnosis despite preceding extensive work-up including [^18^F]FDG PET (change in diagnosis in 32% and 28% of patients) [11, 12]. Two other studies compared the diagnostic value of the two modalities and found that amyloid PET contributed more to the clinical understanding of the patients’ disease and diagnostic changes than [^18^F]FDG PET [8, 16]. Thus, the additional knowledge of the amyloid status of a patient seems to resolve conflicting information provided by clinical assessment and [^18^F]FDG PET. Little is known about the actual incremental diagnostic value of amyloid PET over clinical assessment or [^18^F]FDG PET, which is of interest for formulating effective diagnostic algorithms and for weighting diagnostic information.

Finally, there are very limited, although promising, data available verifying that the diagnostic changes are in fact correct. Ossenkoppele et al. [8] conducted a 2-year clinical follow-up in patients who underwent combined [^18^F]FDG and amyloid PET imaging. The post-PET diagnosis was unchanged in 22 of 23 dementia patients (96%) during follow-up. Sánchez-Juan et al. [16] found a high to very high concordance between post-mortem neuropathology and [^18^F]FDG and amyloid PET (21 of 23 patients, 91%, and 23 of 24 patients, 96%, respectively).

Against this background, in the present study the incremental value of amyloid PET in the differentiation between AD and non-AD disorders in addition to the clinical diagnosis on one hand and [^18^F]FDG PET imaging on the other was determined in a real-life memory clinic population. Both diagnostic paths were reanalysed by independent clinical and imaging experts with incremental access to the data, allowing complete blinding and hence assessment of the proportion of patients with a change in diagnosis in an unbiased manner within each path. Furthermore, the convergence of both independent paths and the agreement with the final diagnosis after an extended prospective follow-up was taken as validation. Finally, the dependence of the incremental value of amyloid PET on age at onset and AUC consistency was also assessed.

## Materials and methods

### Standard protocol approvals, registrations, and patient consents

The local Ethics Committee approved all procedures (proposal no. 318/14) including the prospective clinical follow-up. All participants gave written informed consent.

### Patients

**Fig. 1:**
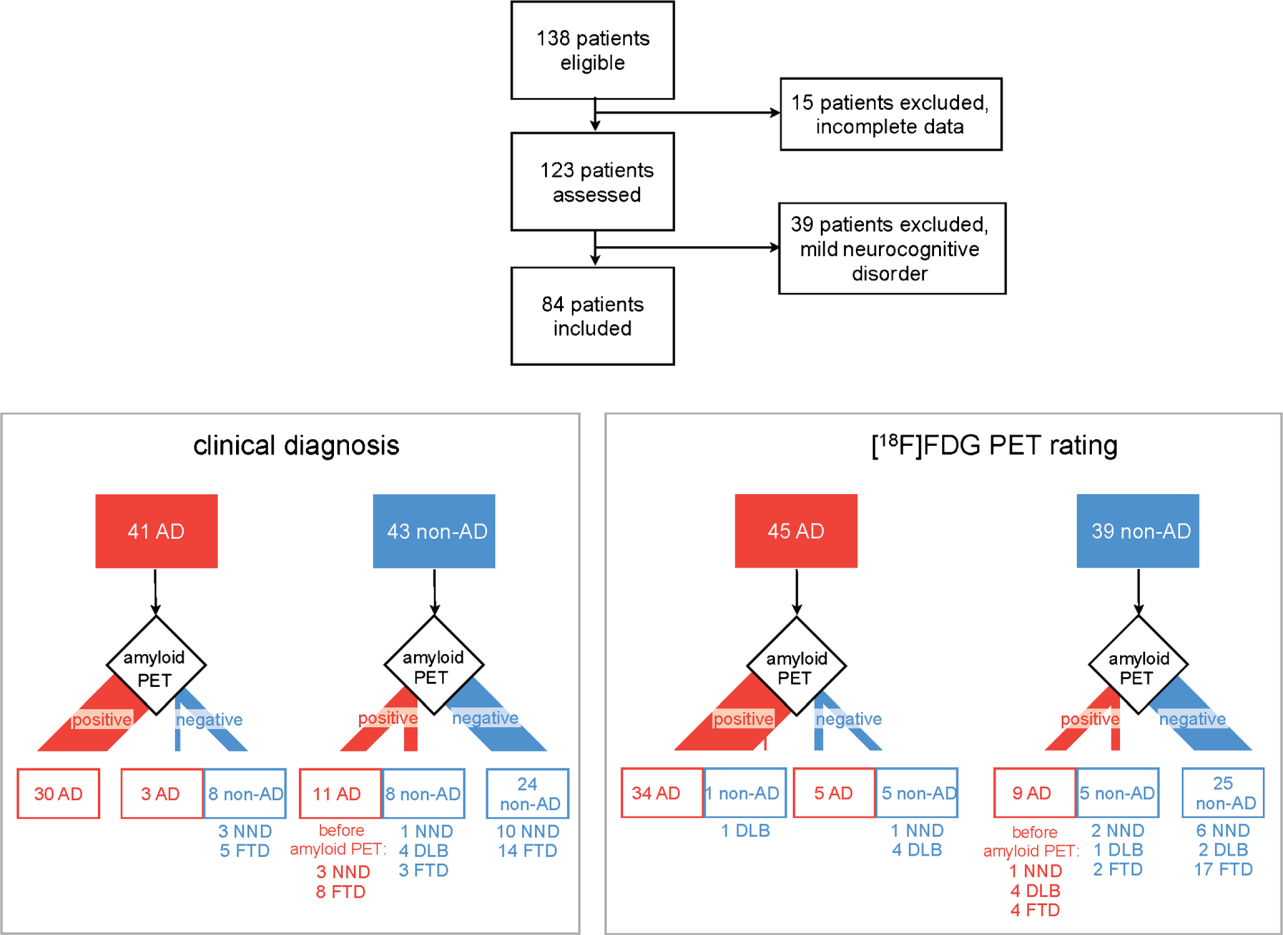
Patient flow and changes in diagnosis. All 84 included patients with major neurocognitive disorder were classified either as suffering from Alzheimer’s dementia (AD, *red*) or non-AD (*blue*) according to the baseline clinical diagnosis (blinded to the PET data, *left lower panel*) or based on [^18^F]FDG PET findings (blinded to the clinical data, *right lower panel*). Changes in diagnosis after disclosure of the beta-amyloid PET results are given. The non-AD group included patients with frontotemporal dementia (*FTD*), dementia with Lewy bodies (*DLB*) and a non-neurodegenerative (*NND*) cause of cognitive impairment

Between May 2009 and August 2013, 138 consecutive patients with cognitive dysfunction of uncertain aetiology (i.e. patients with a complicated clinical presentation) were referred for diagnostic imaging with [^11^C]PIB PET and [^18^F]FDG PET. The majority of patients (>90%) were referred from of the Memory Clinic of the Centre of Geriatrics and Gerontology Freiburg (a tertiary referral centre). All relevant medical charts were thoroughly reviewed by a board-certified dementia specialist (S.H.) in each patient and all relevant clinical information, including neuropsychological assessment, magnetic resonance imaging (MRI), computed tomography (CT), dopamine transporter single-photon emission CT (DAT SPECT), cerebrospinal fluid (CSF) biomarker and genetic testing (except PET imaging), was incorporated into a clinical vignette, which had been developed based on current consensus criteria [3, 19–25]. Clinical vignettes contained neither images nor standardized atrophy measures (i.e. MTA score or Koedam score), but only the clinical MRI and CT reports. At this stage, 15 patients were excluded from further analysis due to incomplete data (mostly external referrals; 123 patients remaining; Fig. 1).

### Clinical ratings

Two other clinical experts (L.F. and T.B.) with long experience in dementia diagnostics filed baseline clinical diagnoses at the time of imaging based on data presented by the case vignettes. First, the diagnoses were dichotomized into mild or major neurocognitive disorder (DSM-5; American Psychiatric Association, 2013). As a result, 39 patients were clinically classified as having mild neurocognitive disorder corresponding to mild cognitive impairment (MCI) according to the classification of Petersen et al. [26], and were excluded from the present analysis. This was done since the present study pursues a validation by specific etiological diagnoses which are of limited applicability in case of MCI (e.g., ambiguous clinical findings at baseline, still unclear situation in case of non-converters at follow-up).

Second, the 84 patients diagnosed with major neurocognitive disorders were allocated to the following diagnostic subgroups based on current consensus criteria (if available): AD [3, 21, 24], dementia with Lewy bodies (DLB) [23], frontotemporal dementia (FTD) [21, 25] and non-neurodegenerative (NND) causes of major neurocognitive disorder (i.e. alcohol-related dementia, vascular dementia, normal pressure hydrocephalus, psychiatric disorders) [20, 22]. The baseline diagnosis was first filed independently by both experts, after which they reached a consensus. In a second session, the results of amyloid PET in all patients were disclosed to the clinical experts and they were asked to adjust their former diagnoses as considered necessary. In this session, only the consensus of the two raters was assessed, since the vignettes provided fairly detailed pictures of individual patients. Therefore, the detailed discussion of individual cases during the first session prohibited further unbiased independent diagnoses by the two raters. For the same reason, a seemingly independent session in which clinical raters had to establish diagnoses with additional knowledge of the [^18^F]FDG PET results was not included (i.e. an unbiased comparison of “clinical plus amyloid PET” vs. “clinical plus [^18^F]FDG PET” was not possible; such a comparison would have required the enrolment of larger, carefully matched patient populations and/or two independent teams of clinical raters). Since clinical differential diagnosis of dementia largely depends on the fairly subjective interpretation of a variety of symptoms, and examination findings and their development, we focused on the consensus as a way to control for subjectivity [27].

### PET readings

The [^11^C]PIB and [^18^F]FDG PET acquisition and processing protocols were as described by Frings et al. [28]. All patients underwent [^11^C]PIB and [^18^F]FDG PET examinations on the same PET scanner. An ECAT EXACT 922/47 PET system (Siemens-CTI) was used in 45 patients with mean ± standard deviation injected doses of 483 ± 95 MBq [^11^C]PIB and 302 ± 19 MBq [^18^F]FDG. A Philips Gemini TrueFlight 64 integrated PET/CT system (TF64, Philips) was used in 39 patients with injected doses of 389 ± 38 MBq [^11^C]PIB and 212 ± 11 MBq [^18^F]FDG. In each patient, [^11^C]PIB and [^18^F]FDG PET scans were acquired within 3 months of each other, and within 1 week in 82% of patients.

PET scans were rated by two experienced readers (P.T.M and R.B.) blinded to the clinical information (except age) after standardized preprocessing by a third independent investigator. Visual readings of [^18^F]FDG PET scans were performed on 30 transaxial slices (4.5 mm thickness) covering the entire brain after anterior commissure–posterior commissure (ac-pc) alignment. Slices were displayed in a standardized fashion (maximum adjustment for optimal display, minimum set to 5% of maximum, monochrome “hot metal” colour scale). The readers also had access to 3D stereotactic surface projections (3D-SSP) depicting each patient’s cerebral [^18^F]FDG uptake and its statistical deviation from uptake in age-matched healthy controls (colour-coded *Z* score −0 to −7; decreases only). *Z*-score maps were calculated using Neurostat/3D-SSP (University of Washington [29]) using default settings, which included scaling to the whole brain, cerebellum, pons and thalamus uptake, and selection of an age-matched normal control group. The Neurostat/3D-SSP output included normalization to all the four reference regions so that the clinical reader was able to ensure that the overall extent or possible regional pronunciation of hypometabolism did not bias the results (e.g. “global” scaling is problematic in a patient with advanced disease, while normalization by the thalamus or cerebellum uptake may be affected by additional vascular lesions or diaschisis; unfortunately, normalization by pons uptake, as often recommended, may suffer from noise and registration errors). We chose this approach to achieve optimal diagnostic accuracy as previously suggested by Frisoni et al. [30].

Given the higher spatial resolution of the Philips Gemini TF64 scanner compared with the Siemens ECAT EXACT 922/47 PET system, scans from the Gemini scanner were smoothed with an isotropic gaussian filter of 5 mm full-width at half-maximum to achieve a resolution that was comparable to that of the ECAT EXACT system and the default Neurostat/3D-SSP normal control database. This approach was validated beforehand by comparing Neurostat/3D-SSP outputs of an independent clinical sample based on a smoothed dataset (compared to default Neurostat/3D-SSP controls) and a nonsmoothed dataset (compared to controls acquired on the Gemini).

In analogy to [^18^F]FDG PET analyses, [^11^C]PIB PET scans were read visually on 30 transaxial slices (4.5 mm thickness) covering the entire brain. Slices were displayed in a standardized fashion (parametric standardized uptake value ratio images with cerebellar cortex as reference, scaled from 0.15 to 3, “cold” colour scale) after ac-pc alignment. Images were processed with PMOD (PMOD Technologies, Switzerland) and Neurostat/3D-SSP (University of Washington [29]).

All [^18^F]FDG images were first rated in random order for the presence of an AD-typical pattern of regional hypometabolism (i.e. involving the temporoparietal, the precuneus/posterior cingulate and possibly the frontal cortex) or other metabolic patterns (i.e. normal; predominant frontal and/or rostral temporal hypometabolism in FTD; predominant posterior cortical hypometabolism including the occipital cortex, possible cingulate island sign and frontal involvement in DLB; other). In a second independent session, all [^11^C]PIB images were rated in random order for the presence or absence of beta-amyloid binding. Finally, in a third session on a different day, combined [^18^F]FDG and [^11^C]PIB images were rated by both raters in random order for a final combined PET diagnosis, i.e. AD or non-AD (normal, FTD, DLB, other). After both investigators had rated all the scans independently, a consensus was reached in each run.

### Reference standard

All 84 patients with a baseline clinical diagnosis of major neurocognitive disorder at the time of imaging underwent an extended follow-up of at least 12 months (mean 2.4 ± 1.3 years). This included routine scheduled clinical visits (26 patients, 31%), visits by invitation (37 patients, 44%; e.g. in patients living further from the hospital or cared for by physicians outside our institutions), and semistructured telephone interviews with the patient and/or the closest caregiver and, if considered necessary, with the care-giving neurologist (21 patients, 25%; e.g. severely impaired and/or immobile patients). The same dementia specialist (S.H.) conducted all examinations by invitation (including neuropsychological testing) and semistructured telephone interviews using parts of the clinical vignette as the questionnaire. This extended follow-up was essential to record disease progression and development of clinical core symptoms (20 patients; e.g. occurrence of hallucinations or parkinsonism in DLB, occurrence of disinhibition or speech deficits in FTD), distinct changes in neuropsychological testing (32 patients) and possibly additional imaging (16 patients; see below) or CSF examination (7 patients).

Final consensus diagnoses as the reference standard were established by an interdisciplinary board (L.F., P.T.M., S.H., T.B.) after an extended follow-up. In accordance with current consensus criteria [3, 21, 23, 25], all available information was taken into account, including the entire clinical follow-up (i.e. before and after PET imaging). The consensus panel had access to all patient files, including CERAD neuropsychological assessment battery results at baseline (in 83 patients) and during follow-up (in 44 patients). Available imaging data included [^11^C]PIB and [^18^F]FDG PET in all patients (in two patients, a second [^18^F]FDG PET scan was performed during follow-up), MRI in 62 patients (53 at baseline, 5 during follow-up, 4 at baseline and during follow-up), CT in 21 patients (17 at baseline, 2 during follow-up, 2 at baseline and during follow-up), and DAT SPECT in 5 patients (4 at baseline, 1 during follow-up). Laboratory findings included CSF biomarkers in 16 patients (9 at baseline, 7 during follow-up) and genetic testing for *PSEN1*, *PSEN2* and *APP* mutations in one patient.

### Consistency with AUC

To evaluate the impact of consistency with current AUC [18], we carefully reviewed the individual case records and classified them as consistent or inconsistent with the AUC. We applied a restrictive interpretation of the AUC as previously suggested by Apostolova et al. [4] and Grundman et al. [15].

### Statistics

The commercial software packages MATLAB R2013b and SPSS 22 (http://www.spss.com) were used for statistical analysis. Patients with the baseline clinical diagnosis of major neurocognitive disorder (i.e. AD, FTD, DLB, or NND) were dichotomised into AD or non-AD disorders. The following analyses were performed:

1. Changes in clinical diagnosis (at the time of PET) as well as [^18^F]FDG PET readings before and after disclosure of the amyloid PET results were assessed and compared using the McNemar test.
2. Agreements between the clinical diagnosis (at the time of PET) and [^18^F]FDG PET diagnosis before and after disclosure of the amyloid PET results were quantified using Cohen’s kappa and compared using the McNemar test.
3. The accuracy of [^18^F]FDG PET diagnosis (alone and after disclosure of the amyloid PET results) and clinical diagnosis (at the time of PET; alone and after disclosure of the amyloid PET results) were calculated with reference to the final consensus diagnosis after an extended follow-up. In the absence of biopsy data, we consider that this diagnosis based on clinical and biomarker information was the most accurate possible life-time diagnosis, as previously suggested [27]. In order to determine the incremental value of amyloid PET, the accuracy of [^18^F]FDG PET alone was compared with the accuracy of the combined [^18^F]FDG PET plus the amyloid PET results using the McNemar test. Similarly, the accuracy of clinical diagnosis was compared with the accuracy of the combined clinical plus amyloid PET assessment using the McNemar test.

Analyses were repeated applying stratification by age of onset (i.e. early onset if age at symptom onset ≤65 years, or late onset if age at symptom onset >65 years) and AUC consistency in the late-onset group (not in the early-onset group since AUC consistency is given in relation to age, AD was always considered as a differential diagnosis).

## Results

### Patient characteristics

Figure 1 and Table 1 summarize patient flow and clinical and demographic variables. According to the baseline clinical diagnosis (consensus of both raters) on PET, 41 and 43 patients were classified as having AD and non-AD, respectively. Inter-rater agreement of the baseline clinical diagnosis was 80% (kappa = 0.59, *p* < 0.001; i.e. moderate agreement [31]).

**Table 1:** Demographic characteristics of the patient groups

Patient group^a^	Sex (f/m)	Age (years)	Symptom duration (years)^b^	Follow-up duration (years)^c^	Education (years)^d^	MMSE score^e^	Consistency with AUC (yes/no)	Hippocampal atrophy (yes/no)^f^
All	41/43	66.9 (8.2)	2.7 (2.2)	2.5 (1.4)	13.5 (3.4)	22.3 (4.6)	69/15	13/44
AD	19/22	65.8 (7.5)	2.7 (2.1)	2.8 (1.3)	13.8 (3.7)	21.2 (4.5)^g^	27/14	8/16
Non-AD^h^	22/21	67.9 (8.8)	2.8 (2.3)	2.2 (1.4)	13.2 (3.1)	23.5 (4.4)	41/1	5/28

Data are given as mean values (standard deviation)

*AD* Alzheimer’s disease, *MMSE* Mini-Mental State Examination, *AUC* Appropriate Use Criteria

^a^According to baseline clinical diagnosis

^b^Before PET

^c^After PET

^d^Available in 73 patients

^e^Available in 79 patients. The MMSE score at the time of PET was not properly archived in five patients

^f^Hippocampal atrophy indicated on MRI in those patients who underwent MRI at baseline

^g^MMSE scores were 20.5 ± 4.2 in patients with early-onset AD and 21.8 ± 4.7 in those with late-onset AD (*p* = 0.354, *t* test), and 22.9 ± 4.6 in patients with early-onset non-AD and 23.8 ± 4.4 in those with late-onset non-AD (*p* = 0.558, *t* test)

^h^Major neurocognitive disorder due to non-AD aetiology

Based on [^18^F]FDG PET, 45 and 39 patients were diagnosed with AD and non-AD, respectively (consensus of both raters). Interrater agreement for [^18^F]FDG PET diagnosis was 82% (kappa = 0.64, *p* < 0.001; i.e. substantial agreement). Interrater agreement for [^11^C]PIB PET diagnosis was very high (99%; kappa = 0.975, *p* < 0.001; i.e. almost perfect agreement). Of 43 patients with a baseline clinical diagnosis of non-AD, 19 (44%) were amyloid-positive, while of 41 patients with a baseline clinical diagnosis of AD, 11 (27%) were amyloid-negative (*p* = 0.11). The proportion of amyloid-positive patients with a diagnosis of non-AD on [^18^F]FDG PET (14/39, 36%) was slightly (though not significantly) larger than the proportion of amyloid-negative patients with an [^18^F] PET rating of AD (10/45, 22%; *p* = 0.16). The frequencies of amyloid-positive non-AD and amyloid-negative AD patients did not differ significantly between baseline clinical and [^18^F]FDG PET diagnoses (*p* > 0.4).

### Change in diagnosis

After disclosure of the amyloid PET result, the clinical diagnosis was changed in 19 of 84 patients (23%; Table 2). The proportion of patients with a change in diagnosis was not dependent on amyloid status (11/49, 22%, amyloid-positive vs. 8/35, 23%, amyloid-negative patients; *p* = 0.91). The amyloid PET result was inconsistent with the baseline clinical diagnosis in 30 of 84 patients (36%), and the clinical diagnosis was changed in 19 of 30 patients (63%). Similarly, knowledge of the amyloid PET result led to a change in the [^18^F]FDG PET diagnosis in 15 of 84 patients (18%; Table 2). Again, this was not dependent on amyloid status (10 of 49, 20%, amyloid-positive patients vs. 5 of 35, 14%, amyloid-negative patients; *p* = 0.48). The amyloid PET results were inconsistent with the [^18^F]FDG PET diagnosis in 24 of 84 patients (29%), and the FDG PET diagnosis was changed in 14 of 24 patients (58%). Access to the amyloid PET result improved interrater agreement for the [^18^F]FDG PET diagnosis (see above) to 92% (kappa = 0.83, *p* < 0.001; i.e. almost perfect agreement), though not significantly (*p* = 0.12, McNemar test). There was no significant difference in the impact of amyloid PET on the clinical and [^18^F]FDG PET diagnoses (all patients, and stratified by amyloid status; all *p* > 0.5, McNemar test).

**Table 2:** Changes in diagnosis after disclosure of the amyloid PET results

	Change in clinical diagnosis (%)	Change in [^18^F]FDG PET diagnosis (%)	Clinical vs. [^18^F]FDG PET
All patients (*n* = 84)	23	18	n.s.
Early onset (≤65 years; *n* = 36)	22	11	n.s.
Late onset (>65 years; *n* = 48)	23	23	n.s.
Early vs. late onset	n.s.	n.s. (*p* = 0.158)	
Late onset, AUC consistent (*n* = 33)	21	24	n.s.
Late onset, not AUC consistent (*n* = 15)	27	20	n.s.
Late onset, consistent vs. not consistent	n.s.	n.s.	

*AUC* Appropriate Use Criteria, *n.s.* not significant

### Agreement between clinical and [^18^F]FDG PET diagnoses

Agreement between the clinical and [^18^F]FDG PET diagnoses prior to disclosure of the amyloid PET results was 62% (52 of 84 patients, kappa = 0.24, *p* < 0.05; i.e. fair agreement; Table 3). After disclosure of the amyloid PET results, agreement improved to 86% (72 of 84 patients, kappa = 0.71, *p* < 0.0001; i.e. excellent agreement). This 24% increase in agreement was significant (*p* < 0.001, McNemar test).

**Table 3:** Agreement between clinical and [^18^F]FDG PET diagnoses

	Disclosure of amyloid PET results		Change (%)
	Before	After	
All patients (*n* = 84)	62 (kappa = 0.24)*	86 (kappa = 0.71)**	24% (chi^2^ = 13.9)**
Early onset (≤65 years; *n* = 36)	64 (kappa = 0.25)	92 (kappa = 0.83)**	28% (chi^2^ = 6.8)**
Late-onset (>65 years; *n* = 48)	60 (kappa = 0.2)	81 (kappa = 0.63)**	21% (chi^2^ = 5.8)*
Early vs. late onset	n.s.	n.s. (*p* = 0.156)	n.s.
Late onset, AUC consistent (*n* = 33)	64 (kappa = 0.17)	85 (kappa = 0.69)**	21% (chi^2^ = 4.0)*
Late onset, not AUC consistent (*n* = 15)	53 (kappa = −0.13)	73 (kappa = 0.40)	20% (chi^2^ = 0.8)
Late onset, consistent vs. not consistent	n.s.	n.s.	n.s.

*AUC* Appropriate Use Criteria, *n.s.* not significant

**p < 0.01, *p < 0.05

### Accuracy with regard to final consensus diagnosis

With disclosure of the amyloid PET results, the accuracy of the clinical diagnosis at the time of PET significantly improved from 71% to 89% (*p* < 0.01, McNemar test), and the accuracy of the [^18^F]FDG PET diagnosis significantly improved from 76% to 94% (*p* < 0.001, McNemar test; Table 4). Overall, the accuracy of the [^18^F]FDG PET and clinical diagnoses did not significantly differ before or after disclosure of the amyloid PET results (*p* = 0.48 and *p* = 0.25, respectively).

**Table 4:** Accuracy of clinical and [^18^F]FDG PET diagnoses before and after disclosure of the amyloid PET results

		All patients (*n* = 84)			Early onset (≤65 years; *n* = 36)			Late onset (>65 years; *n* = 48)				Late onset, AUC consistent (*n* = 33)			Late onset, not AUC consistent (*n* = 15)	
		Before	After	*p* value	Before	After	*p* value	Before	After	*p* value		Before	After	*p* value	Before	After
				Before vs. after^a^			Before vs. after^a^			Before vs. after^a^	Early vs. late^b^			Before vs. after^a^		
Clinical diagnosis	Accuracy (%)	71	89	<0.01	75	92	<0.1	69	88	<0.05	–	67	88	<0.05	73	87
	Sensitivity (%)	68	87		74	87		63	88			36	86		100	90
	Specificity (%)	76	92		77	100		75	88			89	89		20	80
	Positive LR	2.80	10.76		3.20	Infinity		2.50	7.00			3.39	8.14		1.25	4.50
	Negative LR	0.42	0.14		0.34	0.13		0.50	0.14			0.72	0.16		0.00	0.13
[^18^F]FDG PET diagnosis	Accuracy (%)	76	94	<0.01	89	100	–	67	90	<0.01	Before: <0.05 After: <0.1	67	91	<0.05	67	87
	Sensitivity (%)	77	96		91	100		63	92			57	93		70	90
	Specificity (%)	76	92		85	100		71	88			74	89		60	80
	Positive LR	3.15	11.81		5.93	Infinity		2.14	7.33			2.17	8.82		1.75	4.50
	Negative LR	0.31	0.05		0.10	0.00		0.53	0.10			0.58	0.08		0.50	0.13

*LR* likelihood ratio

^a^Significant change in accuracy of diagnosis before vs. after disclosure of amyloid PET results (McNemar’s test)

^b^Significant difference in accuracy between early-onset and late-onset groups (Pearson’s chi-squared test)

### Age-stratified analysis

The age-stratified analysis yielded no significant results concerning the impact of amyloid PET. Whereas the clinical diagnosis changed with comparable frequencies in the two age groups (early onset 22%, late onset 23%), the [^18^F]FDG PET diagnosis was twice as likely to change in the late-onset group (23%) than in the early-onset group (11%) after disclosure of the amyloid PET results (Table 2). However, the difference did not reach the level of significance (*p* = 0.16). Agreement between the clinical and the [^18^F]FDG PET diagnoses did not differ between age groups before disclosure of the amyloid PET results (Table 3). After disclosure of the amyloid PET results, agreement improved slightly more in the early-onset group (+28%, *p* < 0.01 vs. +21%, *p* < 0.05), but the difference was not statistically significant.

In general, diagnostic accuracy (clinical and [^18^F]FDG PET) was slightly higher in the early-onset group than in the late-onset group, but the difference was significant only for the [^18^F]FDG PET diagnoses before disclosure of the amyloid PET results (Table 4). Thus, coming from a lower level of accuracy, the increase in accuracy after disclosure of the amyloid PET results was slightly larger and significant in the late-onset group (not significant in the early-onset group). This led to a very high accuracy in all groups (88–100%), but the accuracy was still higher in the early-onset group than in the late-onset group diagnosed with [^18^F]FDG PET with a trend for significance (*p* = 0.052). Of note, the mean MMSE scores were not different between the early-onset group and the late-onset group (Table 1). Thus, the higher diagnostic accuracy of [^18^F]FDG PET in the early-onset group was not associated with a significantly lower MMSE.

### Stratification according to AUC consistency

Patients with late-onset symptoms were stratified by AUC consistency (whether patients with early-onset symptoms fulfil the AUC in relation to age, AD always considered in the differential diagnosis). The proportions of patients with a change in clinical and [^18^F]FDG PET diagnoses did not differ significantly between groups (Table 2), whereas the agreement between clinical and [^18^F]FDG PET diagnoses (Table 3) was lowest in the 15 patients in the AUC-inconsistent group before disclosure of the amyloid PET results (although not significantly different from that in the 33 patients in the AUC-consistent group. The increase in agreement between the clinical and [^18^F]FDG PET diagnoses after disclosure of the amyloid PET results was similar in the AUC-consistent and the AUC-inconsistent groups, but reached significance only in the AUC-consistent group (Table 3). Finally, the increases in accuracy of the clinical and [^18^F]FDG PET diagnoses (final consensus diagnosis as reference) after disclosure of the amyloid PET results were slightly higher and significant only in the AUC-consistent group (Table 4).

## Discussion

The present study was undertaken to determine the incremental diagnostic value of amyloid PET in addition to clinical diagnosis and [^18^F]FDG PET imaging in differentiating between AD and non-AD disorders in a real-life memory clinic population. The additional knowledge of the patients’ amyloid status led to a change in the clinical and [^18^F]FDG PET diagnoses in about every fourth and fifth patient, respectively. As demonstrated in previous studies, this would be expected to directly affect patient management [5, 7, 9–11, 14]. The correctness of changes in diagnosis after disclosure of the amyloid imaging results is supported by a strong convergence of the two independent diagnostic paths, clinical diagnosis and [^18^F]FDG PET (from 62% to 86% agreement) on the one hand and a strong increase in their diagnostic accuracy compared with the final consensus diagnosis after an extended follow-up (from 71% to 89% and 76% to 94%, respectively) on the other.

In contrast to the baseline clinical diagnosis, 27% and 44% of patients with suspected AD and non-AD were found to be amyloid-negative and amyloid-positive, respectively, which is well in line with the findings of previous studies (16–39% and 29–57%, respectively) [4–10]. Consequently, the overall proportion of patients with a change in clinical diagnosis in the present study (23%) also fits with the results of previous studies (about 20% to 30%, range 9–67%) [4–16]. In comparison with the baseline clinical diagnosis and in line with a slightly higher diagnostic accuracy of [^18^F]FDG PET (see below), the rate of discrepant findings between [^18^F]FDG and amyloid PET was somewhat lower (22% and 36% of patients with a diagnosis of AD and non-AD on [^18^F]FDG PET were amyloid-negative and amyloid-positive, respectively), leading to fewer changes in diagnosis (18%). Of note, the overall concordance between baseline clinical and [^18^F]FDG PET diagnoses with disclosure of the amyloid PET results (54 of 84 patients, 64%, and 60 of 84 patients,71%; Fig. 1) was lower than found by Sánchez-Juan et al. [16] (84% and 82%, respectively), who also performed independent [^18^F]FDG PET and [^11^C]PIB PET readings. However, the latter study included mostly patients with little diagnostic uncertainty from research studies evaluating the usefulness of [^11^C]PIB. Consequently, the overall diagnostic change in that study (9%) was also smaller than in the present study and those discussed above. In contrast to previous studies (Introduction), we did not assess the changes in diagnostic confidence in addition to changes in clinical diagnosis, assuming an increased diagnostic confidence as a prerequisite for changing a diagnosis.

The only moderate accuracy of the baseline clinical diagnosis (sensitivity 68%, specificity 76%) is in line with the findings of a recent large post-mortem study in 919 subjects collected from the National Institute on Aging Alzheimer Disease Centers. Beach et al. [1] found a sensitivity and specificity of 71% each for the clinical diagnosis of ‘probable AD’ and 83% and 55% for the diagnosis of ‘possible AD’. This analysis applies to the criteria of McKhann et al. [2], which also reflects the situation of our baseline clinical diagnoses. These were filed without knowledge of the [^18^F]FDG and amyloid PET results, while MRI (available in 57 patients at baseline) was not augmented by state-of-the-art morphometric analyses. CSF data could only be included in nine baseline clinical vignettes, and exclusion of these nine patients left the results essentially unaltered (data not shown).

Conversely, the value of [^18^F]FDG PET for the differential diagnosis of dementia has been confirmed in several post-mortem studies (for review see Bohnen et al. [32]). Meta-analyses of these studies have indicated a sensitivity of 87–93% and a specificity of 73–81% for discriminating AD verified post mortem from other types of dementia and healthy controls [32, 33]. Whereas the specificity of our baseline [^18^F]FDG PET diagnosis (76%) matches the above data, the sensitivity was somewhat lower (77%). This may be explained by the particular inclusion criteria of the present study (i.e. real-life patients with an uncertain diagnosis at an earlier disease stage), which included a relatively large proportion of patients with amyloid-positive non-AD (36%; see Fig. 1). Taken together, the post-mortem studies suggest that [^18^F]FDG PET yields a somewhat higher diagnostic accuracy than clinical assessment alone, which also held true in the present study (albeit without significance; Table 4). Given that [^18^F]FDG PET would not be performed in daily practice without clinical assessment, a comparison of “clinical diagnosis plus amyloid PET” vs. “clinical diagnosis plus [^18^F]FDG PET” would be of interest. As mentioned above, such a comparison was not possible in an unbiased manner in the present setting with only one team of clinical experts and a limited number of patients. Nevertheless, the present approach provided an important insight into how the novel imaging modality amyloid PET may improve the diagnostic value of [^18^F]FDG PET, the established method. More importantly, the inclusion of the [^18^F]FDG PET-based diagnostic path constitutes an independent validation of the diagnostic path primarily based on clinical assessment.

Apostolova et al. [4] observed a change in clinical diagnosis significantly less frequently in patients with early-onset symptoms than in those with late-onset symptoms (17% vs. 43%). In line with the findings of previous studies (e.g. [34]), the diagnostic accuracy of [^18^F]FDG PET was found to be higher in patients with early-onset symptoms than in those with late-onset symptoms in the present study (Table 4). Consequently, disclosing the amyloid PET results led to fewer changes in the [^18^F]FDG PET diagnosis in patients with early-onset symptoms than in those with late-onset symptoms (11% vs. 23%; Table 2). However, this effect did not reach significance, nor was it apparent in relation to the baseline clinical diagnosis. This difference in relation to the study by Apostolova et al. [4] may be explained by different group compositions (final diagnosis AD in 21 of 24, 88%, and 23 of 36, 64%, patients with early-onset symptoms in that study [4] and our study, respectively). In turn, the results of the present study are in accordance with those of the study by Sánchez-Juan et al. [16] that included fewer patients with an uncertain diagnosis (see above) and did not detect a significant effect of age on the concordance of the clinical diagnosis with the [^18^F]FDG or [^11^C]PIB PET diagnoses. Collectively, these results suggest that the primary role of amyloid PET in young patients with suspected AD is primarily confirmatory [4, 16].

Whereas the early-onset group fulfilled AUC by age, 15 of 48 patients with late-onset symptoms (31%) did not meet the AUC. Other than as expected, the proportions of patients with a change in diagnosis did not differ between the AUC-consistent and AUC-inconsistent late-onset groups (Table 2) and, somewhat surprisingly, agreement between clinical and [^18^F]FDG PET diagnoses was lowest in the AUC-consistent group (i.e. seemingly straightforward cases; Table 3). The former is in line with the findings of the study by Apostolova et al. [4], who also observed no statistically significant impact of AUC consistency on change in clinical diagnosis in a relatively small sample of patients with late-onset symptoms. In contrast, Grundman et al. [15] found that a significantly higher proportion of AUC-consistent patients (62%) had a change in diagnosis than AUC-inconsistent patients (45%), which may partly have been a consequence of the larger sample size (228 patients) and an overall higher frequency of changes due to the inclusion of an indeterminate (syndromic) diagnostic category. As an extension of earlier studies, we also contemplated the increase in agreement between clinical and [^18^F]FDG diagnoses and in diagnostic accuracy, which was only significant in the AUC-consistent late-onset group (Tables 3 and 4; but note the smaller number of AUC-inconsistent patients). Overall, the impact of the AUC appears to have been smaller than expected. It is tempting to speculate that this can be explained to some extent by a false sense of certainty in seemingly straightforward clinical diagnoses given the overall limited accuracy of the clinical diagnosis [1].

We used a multidisciplinary consensus diagnosis after an extended follow-up of 2.5 years after PET as the reference for validating changes in diagnosis after access to the amyloid PET results. We acknowledge that access to the amyloid PET results may have led to an overestimation of its incremental value. However, it has to be emphasized that agreement with the final consensus diagnosis is just one argument that amyloid imaging provides incremental value. Additional support for the impact and additive value of amyloid imaging independent of the final consensus diagnosis at follow-up comes from: (1) the impact on baseline diagnoses of experienced clinicians, (2) the impact on [^18^F]FDG PET diagnoses (including improved interrater agreement), and (3) the resolution of incongruence between clinical and [^18^F]FDG PET diagnoses.

Furthermore, it has to be emphasized that the present approach is in line with current diagnostic criteria for AD [3], which strongly support the use of amyloid imaging for life-time diagnosis of AD. Not least, this recommendation is based on numerous post-mortem studies and antiamyloid trials demonstrating that a sole clinical diagnosis is erroneous in about one third of patients (see, for example, Beach et al [1]). Thus, in the absence of a post-mortem neuropathological diagnosis, we chose a final consensus diagnosis as a reasonable substitute [27], which summarized all available information of our patients and was established in an interdisciplinary consensus. Importantly, amyloid imaging provides just one piece rather than the whole solution to the diagnostic puzzle (for example, the proportion of patients with a change in diagnosis among those with a mismatch between the clinical diagnosis and the amyloid PET result was 63%, and among those with a mismatch between the [^18^F]FDG PET diagnosis and the amyloid-PET result was 58%), of which the extended clinical follow-up is probably the most important piece. Finally, our results concerning the accuracy of the clinical and [^18^F]FDG PET diagnoses without the amyloid PET results are in good agreement with previously reported results based on post-mortem validation (see above), which would not be the case if our reference consensus diagnosis had been affected by major bias.

Our results show that the addition of amyloid PET results to baseline clinical or [^18^F]FDG PET diagnoses leads to a significant increase in diagnostic accuracy (Table 4). Thus, our results in a large population of 84 patients strongly supplement the results of previous studies with extended follow-up periods that confirmed the clinical impact of amyloid PET: unchanged diagnosis after 2 years in 22 of 23 patients (96%) examined using [^18^F]FDG and [^11^C]PIB PET) [8], and agreement between post-mortem neuropathology and [^18^F]FDG PET in 21 of 23 patients (91%) and amyloid PET in 23 of 24 patients (96%) [16].

Our study also raises the question as to which of the two PET approaches should be used in clinical practice (considering that clinical assessment is indispensable). Amyloid PET effectively reduced the proportion of patients with conflicting information provided by clinical assessment and [^18^F]FDG PET (from 38% to 14%; see Table 3), which agrees with the findings of previous studies and the observation that amyloid PET contributes more to the final diagnosis than [^18^F]FDG PET [8, 11, 12, 16]. Consequently, baseline clinical diagnosis plus amyloid imaging reached a high level of accuracy in the present study (89%). On the other hand, the combination of [^18^F]FDG PET and amyloid PET reached an accuracy of 94% with a noteworthy decrease in the false-negative rate from 13% to 4% with equal specificity. Further studies are needed to identify the subset of patients who would benefit from an additional [^18^F]FDG PET scan if an amyloid PET scan is already available.

### Conclusion

The present study relying on highly standardized analyses of real-life patient data and an extended follow-up demonstrated that amyloid PET provides significant incremental diagnostic value beyond clinical and [^18^F]FDG PET diagnoses of AD. Given the high diagnostic accuracy of combined clinical and amyloid PET assessment, further studies are needed to clarify the role of an additional [^18^F]FDG PET scan in these patients.
